# Overcoming plant blindness in science, education, and society

**DOI:** 10.1002/ppp3.51

**Published:** 2019-07-01

**Authors:** Sarah B. Jose, Chih‐Hang Wu, Sophien Kamoun

**Affiliations:** ^1^ Global Plant Council Hamilton Ontario Canada; ^2^ The Sainsbury Laboratory University of East Anglia, Norwich Research Park Norwich UK; ^3^ Present address: Primrose Language Editing Cornwall UK

**Keywords:** botany, education, plant blindness, science communication, zoocentrism

## Abstract

Plants are amazing organisms. They make up around 80% of all biomass on Earth, play important roles in almost all ecosystems, and support humans and other animals by providing shelter, oxygen, and food. Despite this, many people have a tendency to overlook plants, a phenomenon known as “plant blindness.” Here, we explore the reasons behind plant blindness, discuss why some people are relatively unaffected by it, and promote education around plant science to overcome this phenomenon and raise awareness of the importance of plants in the wider community.

Summary

Many people tend to overlook the importance of plants in the biosphere. This phenomenon is described as “plant blindness,” a term proposed 20 years ago to denote the inability of a person to notice plants and/or appreciate their significance. To explore why some people seem immune to plant blindness, we asked plant scientists on Twitter why they became interested in plants. Many replied that their interest developed from early experiences in life or inspiring teachers at school. Others were attracted to the scientific disciplines related to plant science or valued the contribution of plants to global ecosystems and human civilization. Based on these anecdotes and the empirical findings of other researchers, we argue that plants should play a more central role in biological education, from the early years to university and beyond. Furthermore, as plant scientists, we should do our best to raise awareness about the fascinating aspects of plants and their importance in human affairs within the wider community.

## Graphical abstract

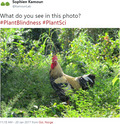

## 1 INTRODUCTION

People tend to overlook plants as living organisms, usually viewing them as unassuming backdrops. This phenomenon, known as plant blindness, also extends to scientists, who often fail to recognize the importance of plants in the biosphere and in human affairs. Even within the biology community, we have encountered colleagues in biomedical fields with a surprisingly limited knowledge of plant biology, from final‐year undergraduate students who did not realize that plants have DNA to researchers who were surprised by the complexity of plants, for example that they have an immune system. In one anecdote, a senior biomedical colleague viewing a video of a *Mimosa* leaf closing exclaimed “it's alive!” Some colleagues even seem surprised that plants matter enough to warrant extensive research and investment in the first place, despite the huge economic and societal impact of crop losses and plant diseases around the world (Savary et al., 2019).

Plants tend to be underrepresented in biology curricula despite being indispensable to all other life on Earth and are hugely prevalent in the biosphere; plants comprise up to ~450 gigatonnes of carbon (Gt C) of the total 550 Gt C of all the Earth's biomass versus just 2 Gt C for animals, most of which is marine life (Bar‐On, Phillips, & Milo, 2018). Surveys have demonstrated that students prefer to learn about animals, and find them easier to recall than plants (Schussler & Olzak, 2008; Wandersee, 1986). This is reflected in the number of undergraduate degrees with a focus on Plant Science in comparison with those specializing in Zoology; for example, in the UK, a search of the Universities and Colleges Admissions Service (UCAS) revealed nine Botany or Plant Science‐focussed Bachelors degrees, while 53 institutions provided Zoology and Animal Biology‐specific undergraduate degrees. Here, we discuss what plant blindness is, and suggest some actions by which we might overcome it.

## 2 WHAT IS PLANT BLINDNESS?

Plant blindness was described by Wandersee and Schussler (1999) as the inability to notice plants in one's environment, recognize their importance or appreciate their unique biological features. One of the major symptoms of plant blindness is the tendency to overlook plants, either because of a lack of knowledge about these organisms, their visual homogeneity, their generally non‐threatening nature or the lack of visual cues such as movement or rapid changes (Wandersee & Schussler, 1999, 2001). Another symptom of plant blindness is the failure to distinguish between the differing biology of plants and animals; the perceived outwardly slow lifecycles and behaviors of most plants mean they do not always captivate our attention in the same way that animals do (New, Cosmides, & Tooby, 2007), leading some to consider plants to be boring (Wandersee & Schussler, 1999, 2001). Ironically, Sanders (2019) pointed out that humans lack the ability to perceive some of the most rapid known plant movements with the naked eye, such as the trapping movements of the carnivorous *Utricularia* genus, raising the question of how to highlight these more “exciting” plant behaviors for the general public.

**Figure 1:**
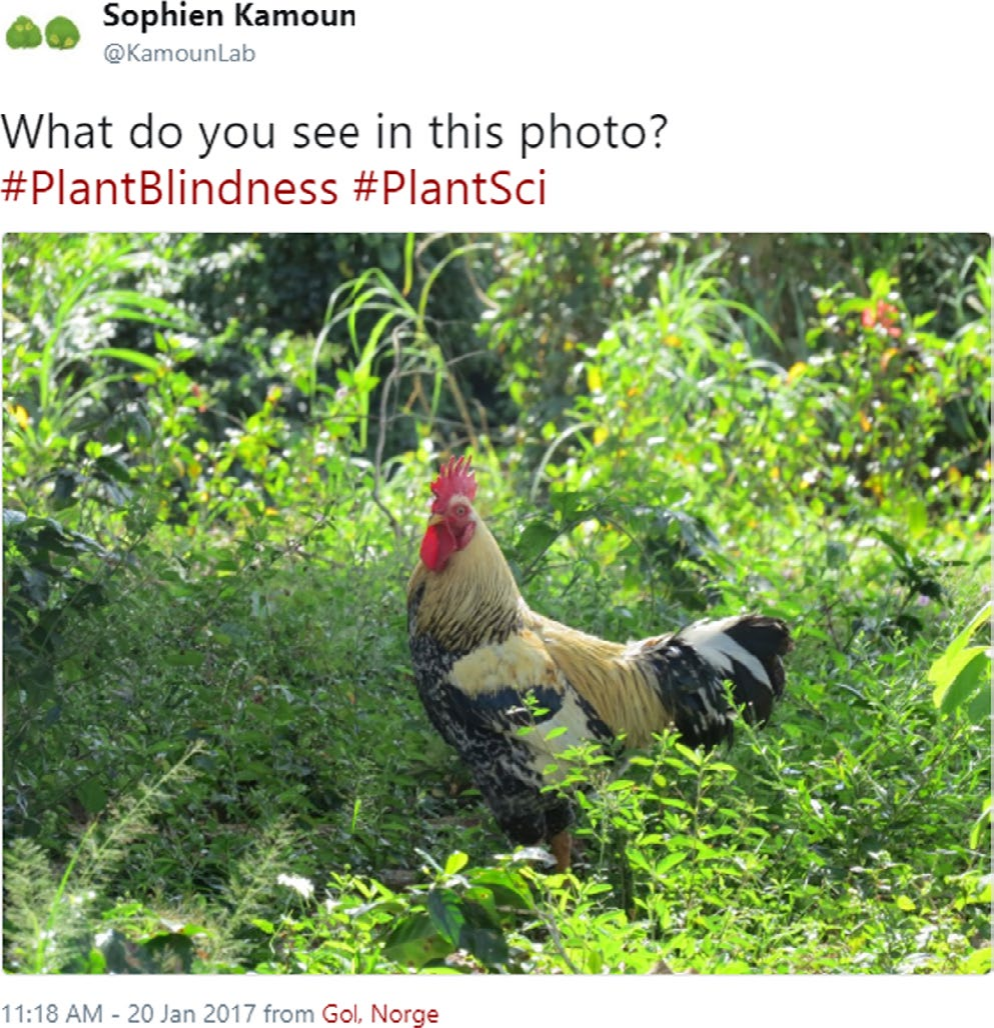
Plants are often ignored in favor of animals. Do you see a rooster or a diverse plant‐dominated ecosystem? https://twitter.com/KamounLab/status/822402983811891200

It is often thought that our intrinsic human nature causes us to overlook the importance of plants. Why is that? Our brains filter out optical signals from the eyes based on our goals, experiences and the potential biological relevance (mating opportunities or threats) of what we observe, meaning that we visually process far less of any given scene than we might expect (Cohen, Dennett, & Kanwisher, 2016). In a study of human attention, New et al. (2007) noted that we are evolutionarily programmed to focus on and respond to animals because as predators or prey they tended to be critical for human survival and their evasion or capture would require a quick reaction. Indeed, Balas and Momsen (2014) reported that participants shown rapid image sequences were much better at accurately identifying those containing animals than plants. Although intimate relationships with plants have developed in some human cultures (discussed by Balding & Williams, 2016), most people may have the tendency to detect and rapidly react to animals, and as a consequence filter out the green “background” of our environment (Figure 1).

## 3 WHY ARE SOME PEOPLE INTERESTED IN PLANTS?

**Figure 2:**
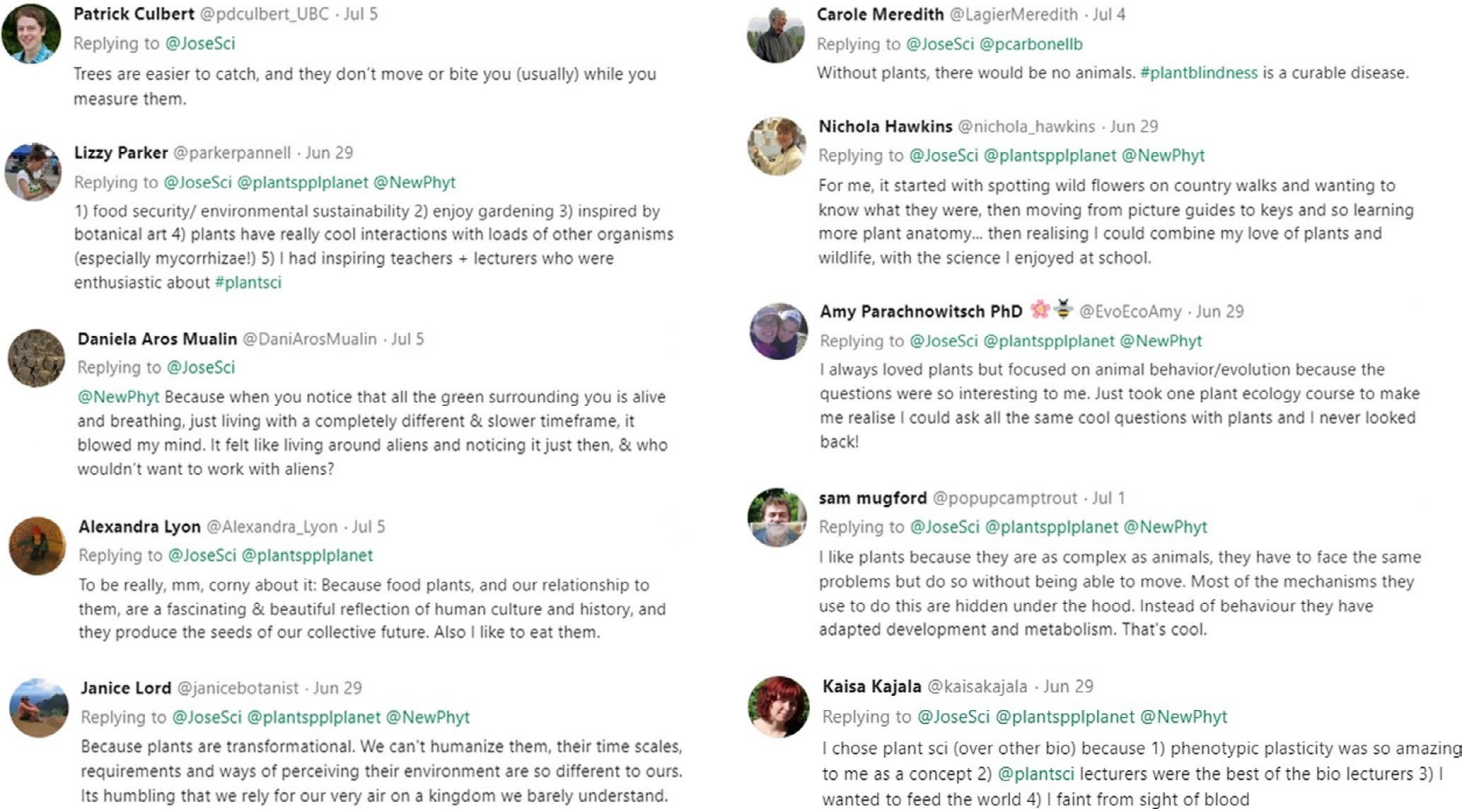
Examples of the many responses to calls on Twitter asking plant scientists why they chose their field of study. The full lists of responses can be viewed under the following Tweets: https://twitter.com/JoseSci/status/1012598291220062209, https://twitter.com/JoseSci/status/1014169141681389568, and https://twitter.com/JoseSci/status/796457569023823872

What differentiates plant scientists and other plant enthusiasts from those who ignore plants? We asked the plant scientists of Twitter why they chose to study the intricacies of plant biology. Many of the respondents reported that their interest in plants developed from early experiences, including growing up on a farm, taking nature walks, or learning from inspirational teachers and lecturers (Figure 2). Others were attracted to other scientific disciplines such as genetics or evolution, and realized they could explore interesting new questions in these fields using plants. Some also mentioned the benefits of studying plants over other research organisms, including their rapid life cycles, capacity to be cultured and grown on a large scale, and ease of genetic analysis, while the many open questions and opportunities for discovery in plant biology drew others to the field. One respondent stated her sudden realization that plants were living on such a different timescale to many other organisms prompted her fascination with plants.

Even if the intricacies of plant biology were not of direct interest, several plant scientists said they chose their field because of the important benefits that plants provide to humankind. Few organisms have such a direct impact on food and ecological security, climate and environmental sustainability, water and nutrient cycles, medicine, and general natural beauty; one respondent eloquently stated that plants “produce the seeds of our collective future” (Figure 2). Understanding these benefits provides immunity against some of the other symptoms of plant blindness listed by Wandersee and Schussler (1999), especially the failure to realize the importance of plants for human existence.

## 4 HOW CAN WE OVERCOME PLANT BLINDNESS?

Early educational experiences providing equal exposure to plants, microbes and animals are crucial for counteracting plant blindness and encouraging future generations of plant scientists. Twenty years ago, Wandersee and Schussler (1999) concluded that plants “have, historically, rewarded our focussed study, observation and investigation,” and emphasized the need to maintain the identity and visibility of botany. Two years later, they hypothesized that early, well‐planned education and interaction with plants is key to overcoming what they describe as the default human condition of plant blindness (Wandersee & Schussler, 2001). We can attest to this, having had the benefit of excellent teachers and mentors whose enthusiasm instilled in us a love of plants at an early age. Several of the respondents on Twitter also described how passionate lecturers converted them from other biological disciplines to plant science, highlighting the importance of high‐quality plant science teaching throughout biological education. To prevent plant blindness in students and encourage them to consider a career in plant science, Schussler and Olzak (2008) proposed that biology teachers should present equal numbers of plant and animal examples to increase student familiarity with, and interest in, plants. Drea (2011) encouraged lecturers to use food security and biodiversity threats to emphasise the importance of plants to our own survival, raising student awareness of the vital services performed by plants. Recently, Krosnick, Baker, and Moore (2018) successfully decreased plant blindness in a group of students by inviting them to grow a plant from seed and monitor its development, while also relating concepts delivered in lectures to these “pet plants.” The authors reported that students had an increased appreciation and attention for plants, with most planning to grow more plants in the future.

In addition to promoting plant science as a career, we should aim to counteract plant blindness in the wider community by raising awareness about the importance of plants in human affairs. This condition is detrimental to society, as plant biodiversity is in a rapid but near‐silent decline that threatens the stability of all of the Earth's ecosystems (Cardinale et al., 2011). Increasing the public appreciation of plants may impact the funding allocated to their conservation (Balding & Williams, 2016), while also highlighting the practical and cultural importance of plants to people who might otherwise never consider it. Our innate animal‐based visual attention can be at least partially balanced by focussing one's attention on plants and their intricacies. Wandersee and Schussler (2001) reported that early hands‐on experiences of growing plants alongside a knowledgeable adult mentor is a good predictor of an interest in and scientific understanding of plants later in life. Whether on Twitter or YouTube, blog posts or magazine articles, TV or radio, face‐to‐face or in lecture halls, we should all share our love of plants as widely as possible, focussing people's attention on the fascinating nature of these underappreciated organisms. We challenge you to do one thing today to spread this message and open someone's eyes to the wonders of plants and plant science.
